# Determinants of Antenatal Care Attendance among Pregnant Women Living in Endemic Malaria Settings: Experience from the Democratic Republic of Congo

**DOI:** 10.1155/2016/5423413

**Published:** 2016-09-15

**Authors:** Célestin Ndosimao Nsibu, Célestin Manianga, Serge Kapanga, Esther Mona, Philippe Pululu, Michel Ntetani Aloni

**Affiliations:** ^1^University Hospital of Kinshasa, Faculty of Medicine, University of Kinshasa, Kinshasa, Democratic Republic of the Congo; ^2^Department of Anthropology, Faculty of Social, Administrative and Political Sciences, University of Kinshasa, Kinshasa, Democratic Republic of the Congo; ^3^Reproductive Health Program, Provincial Coordinator, Kananga, Western Kasai, Democratic Republic of the Congo; ^4^Health District Coordinator, Mbanza-Ngungu, Bas-Congo Province, Democratic Republic of the Congo

## Abstract

*Background*. Antenatal care (ANC) attendance helps pregnant women to benefit from preventive and curative services.* Methods*. Determinants for ANC attendance were identified through a cross-sectional survey in the Democratic Republic of Congo. Sociocultural bottlenecks were assessed via focus groups discussion of married men and women.* Results*. In this survey, 28 of the 500 interviewed pregnant women (5.6%) did not attend ANC services and 82.4% booked over the first trimester. The first visit is positively influenced by the reproductive age (OR: 0.52, 95% CI(0.28–0.95), *p* < 0.04), the educational level (OR: 0.41,95% CI(0.17–0.97), *p* < 0.04), the nearby health center (OR: 0.43, 95% CI(0.2–0.92), *p* < 0.03), and the presence of a male partner (OR: 10.48, 95% CI(2.1–52.23), *p* < 0.001). The barriers to early booking were (i) the cost of service; (ii) the appearance or individual income; (iii) the geographical inaccessibility or distance to health facilities; (iv) social and religious prohibitions; (v) the stigmatization from other women when conceiving in the late ages or young or while still lactating (parity); (vi) the time for waiting for services.* Conclusion*. The early ANC attendance is delayed among poor women with little education and living alone.

## 1. Background

It is recognized that early and regular antenatal care attendance is strongly associated to better maternal and neonatal outcomes because pregnant women who do so fully benefit from its preventive and curative services [1–3]. To reduce maternal and neonatal morbidity and mortality, the World Health Organization (WHO) recommended that pregnant women should receive ANC services at least 4 times starting from the first trimester of pregnancy [4]. These provided services are used for prevention, early diagnosis, and treatment of pregnancy-related problems.

In endemic malaria areas, some high-impact interventions such as the use of impregnated nets, the prompt management of malaria cases and anemia, and the intermittent preventive therapy (IPTp) are implemented to reduce malaria burden in pregnancy in areas with stable transmission of* Plasmodium falciparum* [5].

Malaria is the most common parasitic disease in the world caused by* Plasmodium* and transmitted to humans by an infected female* Anopheles* mosquito. At least 85% of malaria cases in the world are provided by Sub-Saharan Africa and most of them are due to* Plasmodium falciparum* [6]. It is a major risk for more than one billion children under five years and pregnant women. A demographic study conducted in 2007 estimated globally 32 million pregnant women in Sub-Saharan malaria endemic areas and most of them are at risk of the devastating consequences of this disease to themselves and the developing fetus [7]. Unfortunately, there is a nonnegligible number of pregnant women in Sub-Saharan region who do not attend ANC services [8–10].

Through its National Malaria Control Program, the DRC is committed to achieve by 2015 the prevention of 80% of pregnant women. Intermittent preventive therapy (IPTp) is one of the preventive services recommended by the WHO to control this disease. But for this IPTp to be effective it must be administered according to standards that are based, on one side, on its early onset before 20 weeks and, on the other, on the number of recommended doses to be administered. Less than 22% of Congolese pregnant women attend the ANC facilities in the first trimester as reported in 2010 from the MICS survey and therefore most of the targeted women do not benefit from all delivered high-impact interventions [11]. Present recommendations of the WHO impose 4 doses of IPTp to women during their pregnancy [12].

So, this study presents determinants of ANC attendance among pregnant women in four health districts in the DRC and assesses the eventual obstacles and bottle necks which can explain the delayed booking to the ANC services.

## 2. Methods

A research protocol focusing on eventual factors associated to ANC clinics attendance has been developed by the Scientific Committee on Malaria of the University of Kinshasa and carried out on four health districts. Quantitative (cross-sectional) and qualitative (focus groups) surveys were conducted in three different settings (urban, rural, and a mixed urban-rural) of two selected provinces based on the utilization of antenatal care services.

### 2.1. Survey Sites

Data of this study were collected in September 2013 in four health zones (HZ), namely, Demba, Katoka, Tshikaji in the Kasai Occidental province, and Mbanza-Ngungu in Bas-Congo province. The health district of Mbanza Ngungu has 109,480 inhabitants spread over an area of 711 km^2^ (density of 154 inhabitants per km^2^) and 124 villages and 330 streets. It has 27 health facilities including a Reference General Hospital (RGH) and 11 public health centers. Katoka, an urban health district, has an estimated population of 134,794 residents in 9 health areas with 22 health facilities. Tshikaji, a mixed urban-rural health zone, has an estimated number of 112,609 people living in 12 health areas with 13 health facilities including a RGH. Demba a rural district has 301,913 inhabitants living in 22 health areas with 23 health facilities including also a RGH.

The climate of these four health zones is tropical with two seasons, a rainy season which runs from September to mid-May and the other is dry from mid-May to August. The populations of these districts live mainly on subsistence farming; the main agricultural products include maize, cassava, groundnuts, vegetables, and spices, and there is a small livestock.

Malaria, acute respiratory infections, nutritional deficiencies including iron deficiency, sexually transmitted infections, and diarrheal diseases are the most prevalent diseases. The rate of continuous use of ANC services is usually low in these HZ not exceeding 50% on the average of all. Although IPTp is implemented in all clinics and health facilities, the estimated coverage of IPTp-2 remains low in these HZ hardly passing 14%.

Ethnically, Mbanza-Ngungu health zone is predominantly resided by Ndibu and Ngombe tribes, whereas Tshikaji, Katoka, and Demba are predominantly by Lulwa tribes. Each of these tribes speaks different local languages and has local beliefs and traditional lifestyles that differ from those of other tribes.

### 2.2. Tools and Data Collection

Elaborated quantitative and qualitative data collection tools were pretested in 2 health zones, a rural and an urban district in Kinshasa before obtaining the approval of the Ethics Committee of the Public School of Health of the University of Kinshasa. To compare KAP of pregnant women and care providers in different health districts with different ANC coverage rate, a questionnaire was developed based on the generic elaborated by London School of Tropical Medicine and Hygiene. A stratified poll to the household level considered as primary unit has been adopted as sampling method for the quantitative investigation. Households depending on the health center have been identified as the main strata of survey. Thus, lists of districts, cities, and villages assorted by numbers of population have been updated for the pull of the primary units. For qualitative survey, guidelines were elaborated for focus groups of married men and women and for personalized interviews of holders of traditional knowledge and community leaders.

After the training of the field interviewers and their supervisors, collecting quantitative data into 20 clusters took place in 25 selected health areas of the four HZ (10 in Mbanza-Ngungu HZ, 5 for each of the remaining three HZ). In each HZ, two focus groups of 12 married men and 12 married women were organized; personalized interviews were also conducted with community leaders and holders of traditional knowledge.

Data entry was done using Microsoft Office Access and later transferred into STATA statistical software for cleaning and analysis.

### 2.3. Variables

The dependent variables were the timing to start the ANC visits and the number of visits. Independent variables including the distance between a woman's residence and the ANC services, maternal age, marital status, maternal education, ANC motivation, uptake dose of SP, and presence of any barrier or obstacle were identified.

### 2.4. Statistical Analysis

Data were analyzed using both standard descriptive and analytic methods of applied statistics. Frequency distribution of participants across background characteristics and bivariate analysis were performed. The degree of association between each pair of categorical variables was tested using Pearson's Chi-Square (*χ* ^2^). The odds ratio and 95% of confidence interval were performed and the test of association between the outcome and each of the independent variables was considered significant when *p* value showed 5% or less.

### 2.5. Ethical Process

During data collection, participation was voluntary and the potential respondents had first to sign an informed consent form. The interviewer read and explained the content of the consent form to the potential interviewed person. The respondent was also free to read the consent form by herself and ask for any clarification. Then an interview was conducted only if the respondent agreed and signed the consent form to take part in the survey. Data collected remained anonymous.

## 3. Results

### 3.1. Social and Demographic Characteristics of Interviewed Pregnant Women

Five hundred pregnant women were interviewed in the 25 selected health areas.  Table 1 shows their social and demographic characteristics.

The mean age of the interviewed was 26.7 ± 8.6 years, ranging from 14–49 years. Twenty eight of the 500 pregnant women (5.6%) did not know their age. In addition, their distribution according to age groups was as follows: 25/472 (5.3%) were under 18 years old, 80.3% (379) were between 18 and 34 years old, and 68 (14.4%) were more than 34 years old. At least 82% (410) of all these women are married or in a consensual union; 88.6% of the interviewed women had attended school and especially the secondary school for most of them (252/443). Forty-four pregnant women (8.8%) were primiparous.

### 3.2. Organization of ANC Services

The ANC services are provided in the four visited health zones (districts) mainly by qualified personnel: physicians (12%), qualified nurses (60%), midwives (16%), and other personnel (12%). There is no stockout of vaccines (tetanus), iron, folic acid, and anthelmintic for deworming apart from the SP in 6 of the 25 health areas visited. In terms of communication, IEC tools focusing on the early initiation of the ANC and integrated maternal neonatal care are found in only 5 of the 25 health areas (20%).

The ANC services are not easily accessible for 9.4% (47/500) of the interviewed pregnant women who should walk three hours from their house to the health center because they live beyond 15 km away in rural settings. Sixty women (12%) booked for the antenatal visit in the first trimester of their pregnancy.

Data about factors that seem to influence the first antenatal care visit were analyzed (Table 2). This study showed that women at extreme ages (earlier or later) are more likely to book late for ANC than those compatible with reproductive age (OR: 0.52, 95% CI(0.28–0.95), *p* < 0.04). The first visit is positively influenced by the educational level, where those with a higher education level were more than those with primary level or without formal education (OR: 0.41, 95% CI(0.17–0.97), *p* < 0.04), living nearby a health center (OR: 0.43, 95% CI(0.2–0.92), *p* < 0.03), and the presence of a male partner either by marriage or consensual union (OR: 10.48, 95% CI(2.1–52.23), *p* < 0.001).

### 3.3. Why Do They Attend ANC Services?

The reasons why these respondents attended ANC services independently of the period are listed in decreasing proportion: (i) to guarantee the well-being of the mother and her fetus (41%), (ii) to treat pregnancy-related problems (36.4%), (iii) to benefit from preventive medications and vaccines (9.2%), (iv) for the early diagnosis (4.4%), and (v) for better outcomes or a safer delivery (4%).

At least, 150 pregnant women (30%) were recognized to be influenced by social and cultural barriers such as (i) the financial incentives because pregnant women must pay for services; (ii) old clothes or poor attire that make them feel or look ridiculous; (iii) geographical inaccessibility; (iv) social and religious prohibitions; (v) parity; (vi) time for waiting for services. Twenty four interviewed pregnant women (4.8%) did not attend ANC clinics during their current pregnancy or even the last one.

### 3.4. Practice of Intermittent Preventive Treatment (IPTp)

Two hundred and ninety-two women interviewed (58.4%) have knowledge on the prevention against malaria and particularly the IPTp. This preventive treatment was administered to 262 pregnant (52.4%), but only 26.7% received the two doses of SP as recommended by the Congolese National Malaria Control Program. Six interviewed pregnant women have been recognized to use other regimens such as Camoquin® or total extracts of medicinal plants like Manalaria®.

### 3.5. Qualitative Research Based on Focus Group

The four focus groups revealed that married men as well as women are acquainted with the importance to book early for ANC services, but they do not encourage the early booking. They affirmed waiting until pregnancy appeared before attending the ANC services.

To the question “when should a woman start the ANC visit once pregnant?” the majority of the interviewed women or men in these four health zones talked about their experience. A period from the sixth to eighth month was randomly given, evidence that confirms their experience to attend the ANC services late especially in the third trimester. They said the reason they are afraid to announce this particular status is because of fear of wizard's attacks that can get rid of the pregnancy or cause abortion. They mentioned that only women with pregnancy-related diseases should book early.

For the majority of the participants of the focus groups, the ANC visit is seen as an activity associated with spending money for nothing. They argue according to the traditions that “before the second trimester of pregnancy, the fetus is not viable because he is still blood” and believe that there are sociocultural mechanisms such as social incantations or series of rituals or prayers that manage this particular period. The pregnancy is considered as a package that can be closed early or opened when it comes to term; it is recognized as a critical time during which the spirits can disturb the course of the pregnancy.* This is our lifestyle, while praying we must be confident with the will of God and do not book to the ANC services, the fetus is a spirit; whether there are prophecies, we must comply with “Djimi ba mulombela nzambi.”* Instead of spending money, they all affirmed the need to save money for the discharge that must be a party and pay for new clothes and jewelry for the mother and her baby.

A social interpretation was given by all the interviewed relating to the specific activities performed when attending ANC services. The aims of these services are cited: (i) the assessment of the well-being of the mother and her fetus, (ii) the management of diseases related to pregnancy, and (iii) the benefit from preventive interventions such as tetanus vaccine and the early diagnosis of pregnancy.

## 4. Discussion

The objective of this study was to investigate the factors that explain the late initiation in ANC visits in the Congolese community. Nevertheless, it should respond to the specific facts on the supply, use of ANC services, and the various determinants and differences between different tribes residing in these various environmental settings.

The average age of the respondents was 26.71 years with a standard deviation of 8.6 years. This average age is close to that found by Bakouan in Burkina Faso [13]. The population of our study appears relatively young and could probably be receptive to messages promoting the use of prenatal care.

We found a statistically significant relationship between age and the initiation of ANC visit in the first trimester of pregnancy in different health areas surveyed. Women under 18 years and those aged over 34 years seem fewer in the first quarter visit (OR: 0.52, 95% CI(0.28–0.95), *p* < 0.04). The stigmatization from other women when conceiving in late or young age could be a reason to not booking the ANC services early. The MICS carried out in 2010 in DRC reported an observed frequency of 28.4% of Congolese women with less than 19 years of age who have begun childbearing and 3.7% had a live birth before the age of 15 years [11].

Most of the surveyed women in this series live in rural, urban, and mixed urban-rural areas, but there was no significant difference between the three groups in terms of their representation in this study. Nevertheless, we found a statistically significant association between place of residence and the first ANC visit in the first trimester, pregnant women living beyond 15 km from the health center attending late ANC services. This observation was also reported by Bakouan in Burkina Faso [13] and Bonono and Ongolo-Zogo in Cameroon [14]. These pregnant women living away from health facilities were found mainly in rural and mixed urban-rural areas; this clearly indicated that the living environment strongly influences the use of health care and use of services (OR: 0.43, 95% CI (0.2–0.92), *p* < 0.03). But Kyei et al. in rural Zambia [15] found no effect of distance on timing of ANC or number of visits. However, they reported a strong influence of distance to a facility on the quality of the ANC received [15].

This study confirmed several of the factors stressed in the literature such as the educational level that influences attendance at ANC services. Pregnant women at the university level who initiated ANC in the first trimester represent 30.3% against 10.3% at the secondary level and 15.1% at the primary level. Sufficiently educated women seem to be more motivated to take part in early antenatal services than those who were not. These results corroborate those of MICS 2010 [11] where pregnant women with the highest level of education follow the recommendations of the MOH in terms of number of antenatal visits compared to others with a lesser level. Bakouan in Burkina Faso [13], Bonono, Ongolo-Zogo in Cameroon [14], and Birmeta et al. in Central Ethiopia [16] reported the same observation on the relationship between the educational level of the pregnant woman and the attendance of ANC services. At least 11.4% of pregnant women in our series who have no education are less likely to book to these services. But Bouyou-Akotet et al. in Gabon found most frequently those with no education and older among the women who were at the end of their pregnancy with a correct attendance [17].

Regarding marital status, we found that most pregnant women interviewed are married or lived in consensual union with a male partner (82%). These women attended ANC earlier in the first trimester than pregnant women living alone, single, divorced, or widowed. The involvement of the husband seems decisive to book for prenatal care as found by Birmeta et al. in Central Ethiopia [16] and Gross et al. in Tanzania [18]. Matendo also noted the important role of the male partner not only in seeking prenatal care but also for the decision to give birth in a health facility rather than at home [19].

Not a less important fact to note is the influence of gravidity on the early start antenatal care visits as reported by Adegbola while determining the gestational age at antenatal booking in Lagos [20]. He found that primiparous attended ANC services earlier in the first trimester than the others. The diagnosis of pregnancy and especially the joy of living this new experience for the first time had probably influenced the decision of these pregnant women to go quickly to qualified staff. In our study, we did not find any relationship between gravidity and the timing of antenatal booking.

The answers given by the pregnant women about the social concept of ANC and the time to start the first visit are similar to those received at the focus groups of the married men and women. ANC visit has been linked to the activities or tasks that are performed for the well-being of the mother and her fetus. Most of the interviewed women did not mention the first trimester of pregnancy as the period to start booking ANC services, and thus about 88% of them did their first visit beyond the 15th week of gestation. This is consistent with data found by Bakouan [13], Bonono and Ongolo-Zogo [14], and Gross et al. [18].

Social and cultural representations exist in our traditions and seem to influence the attitudes and practices of most pregnant women in our grassroots communities. The fear of wizards and jealously as reported at different focus groups reinforce the KAP of pregnant women to book the ANC services late. Pregnancy is considered a mysterious event that should not be announced early, and pregnant women are convinced of booking late beyond the 5th or 6th month when the secret cannot be kept anymore and she will not suffer from evil spirits and curses. This is reported in several studies carried out in Africa [13–16].

Although these social and cultural practices are currently important and invasive; the emergence of religious practice is another barrier because there are some sects who recommend prophetic antenatal consultations. These facts delay the ANC visits and unfortunately lead to irrational practices such as “*to seal or open the cervix through prayer.*” Despite these facts, as said by these interviewed pregnant women, the greatest barrier is financial inaccessibility, so most of them have suggested making the ANC services free. These observations have been highlighted in some studies carried out in Africa [13, 14, 16]. So, tradition and religious practices were real barriers to early ANC attendance in addition to sociodemographics factors such as particular age groups at both extremes (under 18 and over 34 years), the low educational level (university level consulted earlier), distant delivery sites of the ANC care (beyond 15 km or 3-hour walk), and the inability to pay for the services provided.

Knowledge on the time to start antenatal care visits and the customization of benefits and risks are low as demonstrated above in this series, thus prefiguring a weakness in the development and delivery of specific messages about the early ANC attendance. Existing tools are not updated and many of them are inappropriate for the education of pregnant women according to WHO recommendations. However, guidelines and specific messages were elaborated at the national level, and these data sheets are not always available in field because their distribution depends unfortunately on the formal support received by each health zone from existing partners. It should be imperative to enhance the community awareness about the importance of the ANC [16].

According to WHO recommendations [21], the various education-information-communication (EIC) tools have specific messages. Thus, the first visit is to confirm a pregnancy, calculate the probable term, detect, treat, and administer preventive care, make an emergency plan, and prepare the birth plan, while the other three visits are assigned to tasks such as evaluation of maternal and fetal well-being, excluding gestational hypertension and anemia, taking preventive measures, revision and modification of the emergency plan, and preparations at birth. In this survey, only 10% of the interviewed pregnant women were aware of the need to start the antenatal care early. Most of the pregnant women as well as married men and women claimed that they did not see this early attendance as an opportunity especially if the pregnancy is not affected by any disease. So, it is imperative to improve the dissemination of the educational messages elaborated at the national level to correct and redirect their vision.

Despite its limitations (because the study was conducted only in four health zones of the existing 515), the study was able to explore the most important factors that explain the delay in antenatal care visits in the country. Most pregnant women selected agreed to participate in this survey so the selection bias that could be incriminated was minimized. In addition, interviewers were trained before data collection in understanding the questionnaires in relation to customs and traditions that differ between tribes residing in these different health areas. Mapping survey locations and the choice of households to randomly visit obeyed the strict methodology of a cluster survey. In addition, the visited health areas were categorized into rural, mixed urban-rural, and urban based on the distribution of the MOH recommendations.

## 5. Conclusion

The ANC coverage particularly the attendance to the first visit is low among women who need it more: those who are poor, less educated, living alone, and away from health centers in rural areas. Pregnancy is considered as a natural process of life and sometimes mysterious, and women, families, and communities tend to underestimate the importance of ANC visits. In addition, some of them simply are not sufficiently aware of the risks they face during pregnancy and the benefits they can gain by attending the ANC services early and frequently. We recognize that some sociodemographic characteristics, traditional beliefs and customs, family, and community have a real negative impact on the early ANC initiation in these four surveyed health districts.

## Figures and Tables

**Table 1:** Sociodemographic characteristics of participants (*n* = 500).

Factor	Residence			Total (%)
	Rural	Urban	Urban-rural	
*Age (years)*	26.21 ± 8.3	28.32 ± 8.4	27.82 ± 8.5	26.71 ± 8.6
<18	8	6	11	25 (5.0)
18–34	151	102	126	379 (76.0)
>34	20	17	31	68 (13.6)
Unknown	28	—	—	68 (13.6)
*Parity*				
Primiparous	6	30	8	44 (8.8)
Multiparous	201	95	160	456 (91.2)
*Educational level*				
Primary	108	9	41	158 (31.6)
Secondary	42	94	116	252 (50.4)
Tertiary	1	22	10	33 (6.6)
None	56	0	1	57 (11.4)
*Marital status*				
Married	94	82	146	322 (64.4)
Consensual union	70	9	9	88 (17.6)
Unmarried	37	25	7	69 (13.8)
Divorced	1	7	2	10 (2.0)
Widowed	5	2	4	11 (2.2)

**Table 2:** Relationship between ANC visits and individual factors (*n* = 500).

	ANC attendance		*χ* ^2^	OR	CI 95%	*p* value
	≥14 weeks	<14 weeks				
*Age (years) *						
<18	14	11				
18–34	42	337	**4.61**	**0.52**	**0.28–0.95**	**0.04**
>34	4	64				
*Parity*						
Primiparous	8	36	**1.75**	**1.73**	**0.76–3.92**	**NS**
Multiparous	52	404				
*Educational level*						
Primary	10	23				
Secondary	26	226	**10.56**	**0.26**	**0.11–0.83**	**0.001**
Tertiary	24	134	**4.26**	**0.41**	**0.17–0.97**	**0.04**
*Marital status*						
Married/consensual union	59	351	**12.24**	**10.48**	**2.1–52.23**	**0.001**
Living alone	1	89				
*Residence*						
Nearby or <15 km	50	405	**4.92**	**0.43**	**0.2–0.92**	**0.03**
>15 km	10	35				
